# Could resistance training prevent or improve work-related musculoskeletal disorders among surgeons?

**DOI:** 10.1308/rcsann.2024.0089

**Published:** 2024-10-22

**Authors:** A Vijay, PA Brennan, M Fagbohun, RS Oeppen, D Parry

**Affiliations:** ^1^ King’s College London, UK; ^2^ Portsmouth Hospitals University NHS Trust, UK; ^3^ Park Practice, London, UK; ^4^ University Hospital Southampton NHS Foundation Trust, UK

**Keywords:** human factors, musculoskeletal pain, patient care, wellbeing

## Abstract

Studies have demonstrated the negative impact that work-related musculoskeletal disorders (WMSDs) have on surgeons. These are also likely to affect some allied healthcare professionals such as interventional radiologists. Problems from WMSDs include pain, diminished technical and cognitive performance, and work absence. These could contribute to burnout, to which surgeons are already vulnerable owing to other working practices such as shift patterns and long hours. WMSDs could negatively affect working performance, and lead to poorer surgical outcomes and patient care. Surgeons are at risk of WMSDs of the neck and back that result from fixed and damaging postures while operating. Some have reduced their operation numbers and working days as result of WMSDs. Theatre ergonomics (e.g. table positioning, operating stools and monitors), intraoperative breaks and stretching may improve WMSDs for some.

Strength/resistance training (RT) may be used to prevent or mitigate WMSDs. RT can also enhance general health and concentration, and combat intraoperative fatigue. Low engagement times of moderate-intensity RT of 20 minutes, twice a week, improve neck and back pain from WMSDs. Moreover, RT has been shown to reduce all-cause mortality by up to 15%, increase bone density, improve proprioception and reduce the fear of movement due to pain. Alongside ergonomic improvement and stretching, we recommend RT as an activity to improve general health and WMSDs.

## Introduction

Work-related musculoskeletal disorders (WMSDs), which involve soft and hard tissues, are a main contributor to lost working days, personal distress and lowered professional performance.^1,2^ Surgeons and clinicians from other specialties including interventional radiologists and cardiologists are vulnerable to WMSDs from performing multiple and long procedures. Often, the surgeon is in a fixed posture for extended time periods and this may cause or exacerbate these conditions. Lowered technical or cognitive performance due to a WMSD could have a negative impact on staff retention, technical performance and patient outcomes.

Strength/resistance training (RT) involves weighted exercises that improve muscle strength and endurance.^3^ Types of RT include bodyweight exercises (such as push-ups, pull-ups and squats) and weightlifting. A ‘set’ is a specific RT movement with a given weight for a particular number of repetitions (for example, doing ten squats with a 20kg load). A typical RT routine usually involves multiple sets of exercises targeting different agonistic muscle groups with rest periods between sets.^3–5^ The aim is to progressively increase the repetitions, sets or weight used as strength increases.^3^ RT has been shown to improve postoperative rehabilitation^6^ as well as the symptoms of WMSDs.^4^ Exercise promotes cognitive performance and general health, and is a contributor to reduced stress and improved mental health for surgeons.^1,7^ This paper explores how RT could be used to reduce the impact of WMSDs on surgeons.

## How do WMSDs affect surgeons and other specialties?

Surgeons are not the only group of healthcare workers vulnerable to WMSDs but they may be particularly susceptible. Long working hours and extended periods of holding uncomfortable static positions contribute to WMSDs among the surgical workforce.^8^ Pain and discomfort experienced by surgeons can affect their technical and cognitive ability, and may lead to lost working time due to work absence.^9–11^ Lowered surgical performance is also likely to lead to worse patient outcomes.

WMSDs contribute significantly to surgeon burnout, not only causing ongoing pain and reduced intraoperative performance but also leading to long-term career implications.^12,13^ Pain may be experienced while operating and when not. Around 60% of surgeons have reported chronic back pain.^8^ Epstein *et al* noted an overall career prevalence estimate of 10–20% for WMSD injuries and a 12-month pain prevalence of 40–65%.^8^ The physical burnout of skilled surgeons due to preventable WMSDs is a significant issue, highlighting the need for effective prevention and management strategies to ensure the longevity of surgeons' careers. WMSDs are also an occupational hazard across the surgical specialties.

A quantitative study of 53 orthopaedic surgeons reported that 50% had back and neck pain, with 6 surgeons (11%) stating that they had had to significantly alter their working practice to alleviate the pain.^14^ A third (30%) of the affected group received medical treatment for the pain and the same 30% also reduced the total number of hours that they operated, owing to pain. Fifteen per cent contemplated early retirement because of pain. The WMSDs experienced are likely caused by extended hours spent in non-ergonomic positions. Surgeons stated that the reason for their pain was a combination of long hours of standing, poor posture and holding static positions. Neck pain affected the respondents on an average of 1.73–2.08 days per week and for back pain, this was 2.85 days per week. Respondents stated that it was affecting their operative ability and their life outside work.

Another cross-sectional survey of otolaryngologists reported that 60% suffered from WMSDs, with 65% of these experiencing musculoskeletal pain in multiple body areas.^15^ A further study by Babar-Craig *et al* found a neck and back pain prevalence of 72% among ear, nose and throat (ENT) surgeons, with over 50% attributing this directly to operating.^16^

Poor posture contributes to neck and back pain. A forward head position with hyperextension of the C1–C3 vertebrae and flexion of the C4–C7 vertebrae is an example of a poor posture adopted by some surgeons (including oral and maxillofacial surgeons) while operating. In combination with this neck position, there is often a protracted shoulder, where the acromion is positioned anteriorly in reference to the C7 spinous process. These positions contribute considerably to neck and back pain for the surgeon.^17,18^ In addition, if worn, heavy lead aprons to shield from ionising radiation can become uncomfortable with prolonged use, and may lead to musculoskeletal load and poor posture.

## Current methods for treating WMSDs

Stretching and posture corrections seem to be the mainstay for the treatment of WMSDs.^2,8,14,19^ and these are useful methods for surgeons to adopt. Preventive workplace ergonomics are another way of combating WMSDs. The preventive techniques include simple but effective interventions such as the comfortable positioning of the operating table, using an operating chair or stool, and use of appropriate equipment and instruments. Employing visual adjuncts (e.g. loupes and monitors) is also recommended, where appropriate.^2,8,15^ Some newly designed ergonomic supports have been trialled and have shown relative success in small groups. These new designs are not yet widely used owing to availability and cost limitations.^15^ Ergonomic improvement and intraoperative stretching, either alone or in combination, do not seem to offer a solution to established WMSDs arising from holding static positions while operating or assisting.

Intraoperative stretching by the surgical team alongside taking ‘microbreaks’ every 20–30 minutes of operative time can improve WMSDs and postoperative pain among surgeons.^19^ Simple stretching movements lasting 30–40 seconds have been shown to reduce discomfort/pain in the shoulders, upper limb girdle and hands although these interventions among ENT surgeons did not improve neck and upper back pain, the chief complaint in surgery.^19,20^ However, a bespoke interventional programme for ENT surgeons, designed by Elzomor *et al*, demonstrated improvements for neck and back pain in this group.^19^ An understanding of the problems experienced, together with the postural and anatomical causes, can influence a targeted stretching routine that has superior outcomes.

Prevention of WMSDs is largely neglected among the surgical community. In a national survey of over 300 otolaryngology consultants in the UK, only 31% of respondents were aware of ergonomic principles and only a small proportion of those who were aware reported having applied these principles regularly.^15,16^

## The role of resistance training

RT may prevent musculoskeletal pain, improve concentration and reduce intraoperative fatigue. Regular RT may also improve the general wellbeing of surgeons and other healthcare professionals. Incorporated alongside ergonomic measures, postural changes and intraoperative stretching, we believe that RT is an efficacious adjunct to prevent and treat WMSDs among surgeons.^21–23^

RT is known to prevent musculoskeletal disorders associated with lower back pain, which is one of the most common musculoskeletal complaints among surgeons.^10,23^ A study examining the dose–response relationship between RT and relief for neck/shoulder pain demonstrated that even ten minutes of daily neck and shoulder-focused RT was effective.^24^ No advantage was gained with longer training protocols. Ruivo *et al* showed that moderate-intensity RT, concentrating on the musculature of the shoulder and scapular regions, done for 15–20 minutes twice a week, could improve neck pain.^18^

Considering the necessary amount of RT, Kristensen and Franklyn-Miller found no significant increased benefits for improving chronic back pain were present in completing two sets of RT compared with one single set.^25^ Moreover, there was no additional overall benefit from opting for higher-intensity RT vs moderate-intensity RT for restoring back function and pain improvement. Intensity is a function of weight used for resistance and/or repetition amounts per set. Furthermore, if multiple sets are performed, then the time taken between sets can be a function of intensity, with a lower rest time between sets increasing the intensity (providing the weight and repetition number remain constant). The American College of Sports Medicine has recommended a repetition range between eight and twelve for novice trainers (described as those with no RT experience or who have not trained for more than 6 months).^26^

The permutations that arise from using different repetition numbers, weights and rest times may amount to a confusing scenario for the surgeon who is a beginner at RT. We recommend that advice from a trainer is sought, especially when starting out. The trainer should be experienced in accommodating the goals of the surgeon, and familiar with the prevention and rehabilitation of WMSDs.

Core muscle stabilising exercises have been associated with significantly reduced lower back pain (38.9% total reduction in pain).^27^ These same exercises were shown to improve proprioception, a reduction in fear of movement due to pain and overall lumbar stability.^21,25,27^ It is proposed that RT can enhance central pain inhibitory pathways and lead to exercise-induced hypoalgesia. This mechanism is hypothesised to contribute to lower back pain.^28^ RT could greatly reduce the problems caused by WMSDs. These exercises could also be valuable for those wearing heavy lead aprons for prolonged time periods.

## Resistance training has widespread health benefits

Exercise can improve physical and psychological health, and is considered to benefit overall wellbeing.^7,13,26,29^ Regular exercise is beneficial for reducing cardiovascular disease, diabetes and even cancer. RT is considered to confer these advantageous effects on health. A meta-analysis looking at over 260,000 participants found that RT was associated with a 15% lower risk of all-cause mortality.^29^ RT was also shown to reduce cardiovascular disease, diabetes and total cancer risk by 17%, 17% and 12% respectively, all results being statistically significant and independent of aerobic exercise. By combining aerobic training with RT, the reduction in mortality rose to 40%. Although aerobic exercise alone may not confer the same postural and musculoskeletal benefits as RT, it is evident that having an exercise regime that uses both types of exercise yields the highest overall benefit.

Surgeons surveyed in the UK reported that stroke, heart disease, hypertension and migraine were caused or aggravated by stress^12,13^ but they were unable to exercise owing to lack of time from the demands of a busy surgical career. Many people believe that you need to exercise more than is actually the case in order to achieve benefit. Widespread advice regarding exercise requirement suggests approximately 30 minutes per day of physical activity.^30^ As this quota is unachievable for many surgeons, it is possible that this stops them partaking in any exercise at all, in the mistaken belief that unless the recommended threshold is met, there are no benefits. However, Momma *et al* reported that the lowest risk of all-cause mortality was observed at 82 minutes per week of RT.^29^ This is achievable with less than 15 minutes per day (or slightly longer if not done daily). This information could be motivational for someone looking for the benefits of RT. This lowered amount also makes time management for exercise more realistic and sustainable for some individuals.

In addition to general health benefits, RT helps improve musculoskeletal health. Regular RT can improve bone tissue mass, as per Wolff's law, where a sufficient load needs to be applied to bone to promote appropriate bone growth.^6,25,28,29^ Moreover, RT movements such as deadlifts, back squats and overhead presses, performed at 80–85% of the one-repetition maximum weight (the amount of weight that someone could lift for only one repetition), significantly improved lumbar spine and femoral neck bone mineral density (*p*≤0.001) in postmenopausal women.^28^ Regular RT can improve tendon health, with potential lower rates of tendinopathies. RT increases muscle strength and may offset sarcopenia. Muscles and tendons contribute to joint stability, and the proper functioning of these may reduce the load on the joint.

Core strengthening exercises improve balance and proprioception, and this may improve a surgeon's operative stamina.^8,13,21^ Maintaining or improving overall musculoskeletal and neurological health through RT could be an important contributor to mitigating the effects of WMSDs on surgeons, as well as improving their overall wellbeing.

RT can diminish WMSDs for surgeons, other medical specialists and allied healthcare professionals. It can also improve their overall health by reducing the risk of a variety of pathologies. RT has been shown to reduce non-specific chronic back pain, neck pain and other musculoskeletal issues. It also improves balance and proprioception, and can have a positive impact on general health, pain and operative stamina. The training time required to yield benefit is lower than thought previously, at around 82 minutes per week, making it an attractive method of exercise that is sustainable.^4,5,28,29^

## Conclusions

We recommend a regular RT regime for surgeons as a time-efficient and efficacious form of exercise, preferably (but not necessarily) with aerobic training. A practical and manageable RT programme might consist of up to 20 minutes of moderate-intensity exercises targeting areas commonly affected by WMSDs, conducted twice weekly. Recommended exercises include shoulder presses, latissimus pull-downs and dumbbell rows, performed in 2–3 sets of 8–12 repetitions, with progressive increase in weights as strength increases.^3,5^ For those new to RT, we advise consulting with a trainer experienced in the prevention of WMSDs to ensure proper exercise form and technique so as to maximise the benefits.

Moreover, when RT is combined with ergonomic improvements in the operating theatre, the potential for reducing musculoskeletal issues is further enhanced. By adopting these practices, surgeons are more likely to maintain their physical wellbeing. Pain-free and efficient surgeons are likely to lead to a happier and more productive colleague, improved team dynamics, less time lost from work, longer retention of surgeons in the profession and better patient outcomes.
